# Are clinical trials randomising households to lifestyle interventions to delay cognitive decline feasible? A pilot study to determine the beliefs, preferences, and deterrents for households impacted by dementia based on semi-structured interviews

**DOI:** 10.1186/s12877-022-02941-8

**Published:** 2022-04-13

**Authors:** Maria M. Costello, Christine E. Mc Carthy, Jackie Bosch, Stephanie Robinson, Clodagh McDermott, Michelle D. Canavan, Martin J. O’Donnell

**Affiliations:** 1 HRB-Clinical Research Facility, National University of Ireland Galway, Galway, H91YR71 Ireland; 2 University Hospital Galway, Newcastle Road, Galway, Ireland; 3 School of Rehabilitation Science, McMaster University, Hamilton, Canada

**Keywords:** Caregivers, Dementia, Lifestyle interventions, Behavioural change, Qualitative

## Abstract

**Introduction:**

While lifestyle risk factors are implicated in the development and progression of cognitive impairment, interventional trials of individual participants have yielded unconvincing evidence. We sought to explore the development of lifestyle interventions targeting the household-unit.

**Methods:**

Semi-structured interviews were carried out among eight households affected by cognitive impairment (i.e. member of the household had cognitive impairment). Interviews took place online using a secure, web-based video platform recommended for patient clinician interaction. Interview content was analysed, and important themes identified.

**Results:**

Eighteen participants were interviewed within households, of which eight (one per household) had cognitive impairment and others were spouses or first-degree relatives living in the same home. Several themes emerged; 1) household members without cognitive impairment were more likely to report poor sleep habits, and sleep was perceived to be the hardest behaviour to change; 2) diet generated most interest as a potential lifestyle intervention target as most participants believed there is a strong link with nutrition and cognition; 3) physical activity is challenging to adapt due to lack of motivation and focus when individuals are cognitively impaired. Barriers to study participation, including risk of harm, complexity of intervention and deviation from routine emerged during discussions.

**Conclusions:**

This study identified beliefs and preferences of households towards lifestyle intervention trials. Findings from this study may be used to inform future clinical trial protocols and future qualitative studies should explore acceptability and feasibility of digital intervention applications.

## Introduction

Dementia is a leading cause of disability and dependency worldwide. Although age is a significant risk factor for dementia, this condition is not a normal or expected consequence of getting older. Globally, there are over 50 million people living with dementia with an estimated 10 million new cases annually [1]. Similar to the rest of the world, rates of dementia are increasing in Ireland [2]. Approximately 180,000 people in Ireland are currently, or were previously, carers for a family member or partner with dementia [3] and there are many others unaccounted for who are providing care and support structures in informal ways. There is considerable regional variation in prevalence of dementia across Ireland with some of the highest proportions reported in the West of the country [4], which has an estimated population of 453,109, most of whom are living in rural areas.

Twelve potentially modifiable risk factors for dementia prevention have been identified including physical inactivity, smoking, social isolation and conditions such as diabetes and hypertension [5]. Epidemiological studies have identified poor sleep as a risk factor for dementia and good quality sleep as having a role in maintaining brain health [6]. Sleep patterns change with ageing and multiple lifestyle factors and co-morbidities can contribute to poor sleep hygiene. Pharmacological interventions for sleep are associated with significant adverse effects, particularly in the older population, so trialling a non-pharmacological strategy to improve sleep habits and overall brain health is of interest. Digital cognitive behavioural therapies targeting habits have shown promise for cognitive performance [7] but have not been trialled beyond the individual level. Several large European clinical trials have investigated multicomponent lifestyle interventions for “at risk” populations with varying efficacy on cognitive outcomes [8–11], all of which targeted interventions at an individual level.

Household-level interventions of lifestyle interventions primarily targeting obesity have been trialled within the paediatric population and in indigenous communities (where health councillors made regular home visits to Aboriginal households and supported the setting of dietary and physical activity goals) [12, 13], but there have been no randomised controlled trial (RCT) targeting household-level interventions to delay cognitive decline. There have been small clinical trials enrolling dyads but these have included little information on household structure or caregiver network [14–16] and focused on individual-level outcomes (i.e. individual with cognitive impairment) rather than health outcomes for the entire household.

Overall, individual-level randomised trials of short-term, single domain, lifestyle interventions have not demonstrated a large, meaningful effect. This is thought to be due to several factors, including short follow up periods and methodological limitations including unblinded assessments of outcomes. The Finnish intervention study to prevent cognitive decline and disability (FINGER) [11] randomised individuals to a multidomain intervention (including physical activity, dietary advice and cognitive training), and  demonstrated improvement in global cognitive functioning at 2 years follow up, and from this trial the World-Wide FINGERS network has been established bringing together global trials with methodological features in common with the original FINGER study [17]. Beyond this, consideration should be given to household level interventions (where the unit of randomisation is the household rather than the individual) to determine if a greater, sustainable effect on cognition can be achieved. Social contact has been identified as a protective factor in delaying dementia, and engaging with family regularly plays a prominent role [5]. Household level interventions may confer benefit beyond the individual with cognitive impairment, with collateral benefits for all household members. With many family members providing informal care duties for the individual with cognitive impairment, they may be at risk of emotional and physical strain leading to adverse health outcomes, physical injury, change in immune response and lack of engagement with their own preventative health strategies [18, 19]. Apart from cognitive and physical health benefits for the participants, targeting a household may improve feasibility and long-term sustained change in lifestyle, by changing the culture within the home. Given the prevalence of dementia, any strategy shown to benefit overall brain health is of use, and if individuals feel they are taking control to prevent further cognitive deterioration this may be, in itself, of benefit [20]. All members of households affected by dementia may, therefore, benefit from targeted lifestyle interventions.

## Study aim

The aim of this study was to explore the feasibility and attitudes towards introducing lifestyle-based interventions in households affected by dementia. Our aim was to better understand the beliefs of the household members around lifestyle factors such as sleep, diet and physical activity and their link with dementia; what challenges households affected by dementia might face in changing lifestyle factors; and how feasible it would be to sustain change among all household members.

## Methods

### Design

We collected data from participants using semi-structured interviews (SSI). The qualitative method of thematic analysis [21] was used. This method was chosen given that it is the most common form of qualitative analysis and provided a flexible approach given that the interviews were adaptable to the participants. We explored the opinions and beliefs of household members on pre-determined topics of interest and spent time exploring, in depth, any factors which were of importance to any household member.

### Ethical approval

Ethical Approval for this study was obtained from the Ethics Committee at University Hospital Galway (UHG).

### Participants

Two investigators (MMC and CEMcC) identified, screened, contacted, obtained verbal consent, and interviewed household members. Study participants were identified prospectively though the dedicated Memory Clinic service, Department of Geriatric Medicine, UHG between October 2020 and May 2021. The inclusion criteria for this study was broad, with consecutive patients referred to the clinic, living in a community-dwelling household with any level of impaired cognition (ranging from mild cognitive impairment to dementia), eligible for invitation to participate, along with their household members. Information regarding the study was given to all potential participants and follow-up phone contact was made to explain the aim of the study, and to reiterate that participation was voluntary. Written consent was obtained from all household members who participated in the study.

Interviews were carried out among each household separately, to ensure all household members had the opportunity to give their opinion. In light of the COVID-19 pandemic, interviews took place online using the secure, web-based video platform recommended by the Health Services Executive (HSE) for patient-clinician interaction. One household completed the interview via telephone due to internet connectivity issues and one household elected to be interviewed face-to-face at the request of the individual with cognitive impairment.

### Sample size

Eight households in total were recruited for this study. Unlike quantitative research where statistical guidelines exist for sample size calculation, there remains practical uncertainty around sample size justification in qualitative research [22]. Our initial goal was to meet thematic saturation (whereby further interviews would have yielded no new themes) [23] however there were several practical factors which influenced the size of the sample. These included difficulty in getting household members to be available at the same time to complete interviews together, challenges in prospective recruitment due to interruption of services during peak waves of the COVID-19 pandemic, and a limited pool of potential participants given many individuals attending the memory clinic were living independently. Although the sample size appears small, and it was difficult to judge when thematic saturation was reached, detailed information was gathered, and similar concepts were repeated among households, suggesting appropriate thematic conclusions could be made in this small, in-depth, study.

### Data collection

Demographic details including age, sex and relationship to household members were collected at time of consent, along with the most recent neurocognitive test results (e.g. Montreal Cognitive Assessment Score) and functional assessments performed within the memory clinic. In advance of the interviews taking place, a standardized interview guide was developed for use by investigators. During each interview, one researcher took the lead as interviewer and the other acted as moderator and facilitated if any technical challenges arose for participants. The interview guide was developed via research group consensus. Questions were developed around pre-determined topics and specific follow up questions were used to explore themes and opinions that participants volunteered. The pre-determined questions were devised based on research group experience of discussions on lifestyle habits and interventions within the memory clinic, by both patients and their caregivers. If there was a particular area of interest for participants, this was explored in further detail.

Open-ended questions were aligned according to the following subtopics; Attitudes towards lifestyle factors; beliefs towards sleep, diet, exercise in cognition, specifically how they contribute to dementia, and the ability to modify them; barriers and challenges to individuals/households in changing lifestyle behaviours, self-efficacy and feasibility in changing lifestyle behaviours and willingness to participate in trials exploring lifestyle factors as a household (Table 1). During the course of the interview, participants were shown a video of a sample behavioural intervention targeting sleep called Sleepio to explore attitudes towards digital interventions [24]. No formal measurements of sleep, diet or physical activity were taken as part of this study.

**Table 1:** Interview Topics for all household members

Topic 1	General attitudes to sleep, diet, physical activity and cognition.
Topic 2	Barriers & challenges to lifestyle change
Topic 3	Self-efficacy/feasibility of changing lifestyle behaviours
Topic 4	Willingness to participate in household level clinical trials

Interviews were recorded with permission of all participants. This was to allow for detailed analysis of the responses to be performed. Any identifying information disclosed during the interview process was not included in the interview transcriptions.

### Data analysis

Recorded interviews were transcribed in full and were analysed by two investigators, MMC and CEMcC. Interview notes were reviewed to look for patterns across households. A codebook was created informed by the questions posed to households, being further informed by the patterns which emerged during the interviews. Inter and intra household disagreements and agreements were noted. Quotations, when relating to a specific topic, were recorded where relevant, and could be contributed from any household member. Key phrases were quantified during the analysis with frequency taken as a marker of importance among households. Codes and quotes were grouped into themes, which were then revised by group consensus, and used to inform the narrative content of this paper.

### Reflexivity

This research was based within the clinical setting of a West of Ireland memory clinic to improve the needs of the population in this large geographical region. The assumption is often held that this population would have little access to, or support to engage in, digital interventions. In addition, further assumptions would include that this older, cognitively impaired population would have little interest in engaging in clinical trials of lifestyle interventions. The aim of this study was to assess if this was truly the case and to provide a clear evidence base of this population for a future clinical trial protocol.

Within this study the interviews were carried out by clinical research fellows (MMC and CEMcC) and both needed to consider how their previous clinical experience and professional knowledge would impact on the interview process. This was taken into account while concluding findings from the data collected, by discussing as a group if the clinical background of the interviewers could have affected the direction of the interviews. To mitigate this bias, the semi-structured format was chosen to allow open discourse and participants (in particular those with cognitive impairment) to lead, allowing them to focus on perspectives on lifestyle habits of most importance to them. To ensure that a range of perspectives were obtained, the households included varied in sex, urban versus rural location and relationship to other household members. As a research group it was discussed if those recruited would be appropriately representative and subject to selection bias. To reduce this risk households were recruited prospectively from the clinic and the inclusion criteria remained broad.

During the reporting of the data, three team members (MMC, MDC and MO’D) met on a regular basis to discuss the data responses that were emerging, if the interview guide questions were effective and if further households should participate, particularly with the limitations of the COVID-19 pandemic. The research group also closely met during the data analysis phase to address any emerging issues. We were sensitive to the fact that all authors had a clinical background in interacting with individuals and families affected by cognitive impairment, but from diverse perspectives, and levels of experience, which helped shape both a well-rounded interview guide and study findings.

## Results

### Demographics/household characteristics

In total, eight households participated in the semi-structured interviews with a total of 18 participants. The characteristics of the households are outlined in Table 2. Of the 8 individuals with cognitive impairment, 75% (*n* = 6) were male, with a median age of 78.5 (range 71-87) years and all had cognitive test scores that were considered impaired (median MOCA14/30 [range 10-23]). Duration of symptomatic cognitive decline ranged from 1 to 5 years, 2 of 8 required assistance with personal activities of daily living (PADL) and all required assistance or were dependent for instrumental activities of daily living (IADL). All were living in the West of Ireland. In terms of the relationship to the person with dementia, among the 10 household members that participated, 50% (*n* = 5) were spouses, 30% (*n* = 3) daughters, 10% (*n* = 1) a son and 10% (*n* = 1) a daughter-in-law.

**Table 2:** Demographic details of study participants

Household	Sex of PWD	Age of PWD (yrs)	Neurocognitive Disorder	Cognitive Test Results	PADL	IADL	Sex of other Household member(s)	Relationship to PWD	Formal Home Supports
**1**	Male	71	Alzheimer’s Disease	MoCA 13/30	I	A	Female	Wife	None
							Female	Daughter	
**2**	Male	73	Mild Cognitive Impairment	MoCA 23/30	I	A	Female	Wife	None
**3**	Male	78	Mixed vascular and Alzheimer’s Disease	MoCA 15/30	A	A	Female	Daughter-in-law	None
							Male	Son	
							Male	Grandson	
**4**	Female	87	Alzheimer’s Disease	MoCA 19/30	I	A	Female	Daughter	None
**5**	Female	79	Mixed vascular and Alzheimer’s Disease	MoCA 10/30	A	D	Male	Son	Home Help
**6**	Male	86	Mild Cognitive Impairment	ACE-III 73/100	I	A	Female	Wife	None
**7**	Male	80	Lewy Body Dementia	MoCA 14/30	I	A	Female	Wife	Home Help
							Female	Daughter	
**8**	Male	77	Vascular Dementia	MoCA 14/30	I	A	Female	Wife	None

*PWD* person with dementia, *ACE-III* Addenbrooke’s cognitive examination III, *MoCA* Montreal Cognitive Assessment, *PADL* Personal Activities of daily living, *IADL* Instrumental Activities of Daily Living, *I* Independent, *A* Assistance Needed, *D* Dependent

### Responses to interview topics

#### General attitudes to sleep, diet, physical activity and cognition

The majority of participants rated their sleep as good or very good (*n* = 13), two persons with cognitive impairment described their sleep as poor and three household members without cognitive impairment described their sleep as poor or very poor. Of the households where members reported very poor sleep, the person with cognitive impairment within the household described their own sleep as very good. All interviewees, including those with cognitive impairment, reported sleep as being important or very important to themselves.

Six households felt that sleep was important in protecting brain reserve and memory function. One daughter commented “If you don’t sleep your brain isn’t given time to process or charge”. Two households did not place much importance on the relationship between sleep and cognitive function, with one spouse noting that her husband [with cognitive impairment] always had much better sleeping patterns and “his memory is worse”. Of the households who felt it was important, they noted that reduced sleep resulted in mental slowing and difficulty concentrating; “I’m not as sharp as I’d like to be if I don’t sleep well” [daughter-in-law, Household 3]; “when you’re that tired….you can’t think straight”[person with cognitive impairment, Household 4]. All households reported poor concentration if insufficient sleep the night before.

Six participants reported getting 7-8 h of sleep per night, three reported greater than 8 h of sleep per night and half of participants (*n* = 9) reported less than 6 h of sleep per night. Only one household reported that all members had similar sleeping habits; other households reported variation in time to bed, time to getting up and daytime napping. In one household, the person with cognitive impairment had visual hallucinations at nighttime and, although he did not report poor sleep, this lead to sleep disturbance for other household members who would wake in response to his interactions and need to re-orient him on a regular basis.

Interviewees self-reported having a healthy or very healthy diet (*n* = 17) with one household member reporting poor diet due to snacking and “comfort eating”. Perceptions of healthy diet included eating fresh fruit and vegetables, consumption of fish 1-2 times per week, and reduced intake of red meat. In Household 1, the spouse had been recently diagnosed with celiac disease, which had resulted in a switch to gluten-free meals for the entire household. Other than one household, all reported having the same dietary intake, at mealtime, within the household. In Household 3, the son adopts a vegan approach intermittently, which means two separate dinners are prepared. In six households, the person with cognitive impairment was dependent on other household members to decide which food was in the house and for meal preparation while in Households 4 and 5 these responsibilities were shared among members. No household reported that the person with cognitive impairment was the main meal preparer.

All households reported that diet was important or very important in protecting brain reserve and memory function: “you hear about omega and cod liver oils…..we specifically eat fish regularly as it is supposed to be good for brain” [Household 2]. Most households (*n* = 6) were uncertain of the exact mechanism through which diet and cognition might be linked, but were able to contribute specific foods they felt were good for cognition with fresh fruit and vegetables rated highest, followed by fish. Household 1 commented that alcohol was “definitely bad for the memory”. No household named a specific dietary type (e.g. Mediterranean). Household 2 commented “I don’t like the word diet, it sets you up to fail. I prefer healthy eating or way of eating” [spouse].

Physical activity patterns varied among and within households. Most interviewees reported walking as their main form of physical activity. Households 1, 6 and 7 reported “lack of interest” in physical activity among the individuals with cognitive impairment and they were less active than other members within households. Household members without cognitive impairment reported higher levels of physical activity, with many spouses achieving a walk of 30 min 4-6 times a week. Others reported participating in yoga, swimming and outdoor exercise classes and in Households 6 and 8 the spouses spent a lot of time gardening. Six households reported enjoying exercise, one household did not overly enjoy exercise and one household reported having “to endure” physical activity. Half of households participated in physical activity together (mainly walking), while the remaining half preferred to exercise separately.

All households reported that physical activity was important, with two households reporting that it was very important, in protecting brain reserve and memory function. Households reported many different ways in which physical activity played a role in preserving cognition; “I see exercise is the pump to keep the brain going” [daughter-in-law, Household 3]; “Exercise is an activity, you need to have your wits about you…being safe…concentrating on what is around you” [person with cognitive impairment, Household 2]. Six households reported physical activity as being important to relax and for stress relief to help with cognitive function. Other reported benefits included environmental change, being outdoors and socially interacting with others.

#### Barriers & challenges to individual/household in changing lifestyle factors

Three households stated they would be interested in changing their sleep habits using a lifestyle intervention, one household was potentially interested, and the remainder were not currently interested. Half of households felt it would be easier to improve sleep quality at an individual level, compared to improving it among the whole household, while the remainder had no opinion on the matter. Household 7 had the most uncertainty around this as, due to Lewy body dementia, visual hallucinations were very prominent for the individual with cognitive impairment at night-time. The main barrier to changing sleep quality at a household level was reported to be the inter-individual variation of sleep patterns within the household.

The majority of households (*n* = 7) reported that it would be easier to change the dietary habits of the entire household, rather than that of an individual alone. Most reported no concerns around a potential dietary change, however the individual with cognitive impairment from Household 8 wished to be certain any dietary reccomendations would not interfere with his medications. Cost or sourcing produce was not a concern among any household.

Three households felt it would be easier to improve physical activity levels as a household unit than at an individual-level, three households felt it would be easier to improve at an individual level and the remaining two households felt it would depend on the type of physical activity and the routine of the household. The main barriers to implementing a new physical activity programme were risk of physical injury and falls, followed by change in routine.

Household income was discussed with all households to determine if any participants had concerns about financial limitations in implementing behavioural change. There were no concerns disclosed, with many having good access to quality food produce and living nearby green spaces where physical activity could easily take place.

#### Self-efficacy and feasibility in changing lifestyle factors

Half of participants reported that it would be difficult to change sleep habits, and that any change would be difficult to sustain long-term, while the remainder had no opinion of this. In comparison, all households felt it would be very feasible to change and sustain dietary habits, especially if there was regular support, recipe ideas and that the foods recommended tasted nice. Five households felt it would be realistic to change physical activity levels among all household members while the remainder felt it would not be realistic or were uncertain. When asked about methods to overcome barriers to improving physical activity, clear instructions, regular support and enjoyment were mentioned most frequently. Household 3 commented it would help “if people were taught self-compassion and awareness of self…journey would be possible for anyone at any age” [daughter-in -law].

Six households felt that of the three specific lifestyle factors (sleep, diet and physical activity), diet would be the easiest lifestyle habit to change in a household, while the remaining two ranked physical activity [Household 2] and sleep [Household 3] as easiest to change. Half of households felt it would be difficult to try and change all three lifestyle habits in combination, with the remainder uncertain if it could be achieved.

#### Willingness to participate in trials exploring lifestyle factors as a household

##### Attitudes to digital interventions

Five households reported that an online intervention would be easy for them to use, especially if via smartphone or tablet application. The remaining three households stated they would find it difficult with main concerns being computer literacy and need for technical support from children outside of the home. Five households reported no issues with internet connectivity while the remainder reported intermittent difficulties with Wi-Fi. All had internet available in their homes. Seven households reported that at least one member would have no issues with typing, with one household reporting the person with cognitive impairment would be especially limited as he had no experience of using computers or typing on a smartphone. Only one household (Household 6) reported that they could be limited due to hearing issues.

##### Participation in future trials

Six households were very interested in participating in a future clinical trial to improve cognition by introducing sleep, dietary and/or physical activity interventions, and which did not involve any pharmaceutical agents. The remaining two households stated they were “potentially” or “maybe” interested. With regards to trial outcomes, fitness, stimulation of memory and improvement in blood pressure was most frequently mentioned as being of importance to interviewees, followed by laboratory measurements of blood glucose and cholesterol levels. Regular support from trial staff, reminders and regular education were mentioned as ways to ease participation and avoid dropping out of the trial, with one household mentioning that virtual calls and assessments would make it easier to participate due to their rural location.

## Discussion

In this study we used semi-structured interviews to explore themes relating to the beliefs, preferences, and barriers of lifestyles (diet, sleep and physical activity) among households where a member had cognitive impairment. These lifestyle factors are similar in complexity in terms of measurement, intervention and optimal dose. There has been a great interest in diet as a modifiable risk factor for dementia. Until recently, there has been limited evidence to suggest any form of additional dietary supplements play a role in dementia prevention [25–28] however the recently published LipiDiDiet clinical trial, randomising participants with prodromal Alzheimer’s Disease to Fortasyn Connect (Souvenaid) or placebo,  demonstrated promising improvements in cognition, functional outcomes and cerebral atrophy within the intervention group [29]. Beyond this, there is a shift to explore the role of whole dietary patterns. The World Health Organisation recommend the Mediterranean diet for delaying dementia,  although the evidence remains limited, with small effect sizes on cognitive performance demonstrated [30]. It has been proposed that adhering to this diet may improve cognition through several biological mechanisms including reducing oxidative stress and neuroinflammation, improving metabolic control and minimising incidence of diabetes, hyperlipidaemia and coronary heart disease [31]. A recent systematic review identified that adherence to such diets in mid-life has promise for neuroprotection in later life [32]. Buckinx et al. applied the GRADE approach (Grades of Recommendation, Assessment, Development, and Evaluation) to determine recommendations for the preferred diet to prevent or to treat cognitive impairment, and although diet was determined to have an important role, specific guidelines could not be determined from the evidence to date [33]. Further randomised controlled trials targeting diet of at-risk populations, with sufficient follow up periods, are necessary to truly determine impact on cognition.

Physical activity has been identified as a key target for maintaining good cognitive function. There is still uncertainty around the optimal dose of physical activity for brain health, with lack of clarity on how this is best measured [34]. There are several factors to consider when investigating the effect of physical activity on long term cognition including stage of cognitive dysfunction, associated functional impairments, intensity of activity and type of exercise. Physical activity which improves cardiorespiratory fitness levels is thought to be potentially beneficial to delay cognitive decline among healthy older adults. This has been a challenging area for comparison given differences in cognitive testing used and there is uncertainty around which specific areas of cognitive function benefit the most (e.g. visuospatial skills, motor function, cognitive speed) [35]. The cognitive benefit of physical activity in older adults has been reviewed extensively [36–38], but clear clinical consensus is difficult to determine as many trials are limited in size, have inappropriate control populations, have short follow-up periods and use cognitive measurement tools that may not reflect clinically meaningful change. In a systematic review of randomised clinical trials published in September 2021, Liu et al. determined that older adults gain benefit from physical activity interventions in terms of mobility and physical functioning but overall there was no clear evidence of improvement in cognitive functioning [39]. Further research is required, not only to determine type and duration of physical activity, but also if benefits vary by dementia type.

Interrupted sleep and greater prevalence of sleep disorders are common features of dementia and there is growing evidence that poor sleep may be a risk factor for dementia. There is a U-shaped association, with both sleep deprivation and excessive sleep associated with increased risk of mild cognitive impairment and dementia [5]. The mechanism for this association remains unclear, as it is uncertain if extremes in sleep duration are a risk factor or an early consequence of cognitive impairment. Biologically, shorter sleep durations have been associated with neuronal pathway disruption and amyloid-β accumulation [40] but there have been no similar causal mechanisms identified for longer sleep durations [41]. New onset disturbed sleep in older age may be a very early feature of a dementia process and therefore may be a suitable time-point to intervene. Given the harm, including increased risk of falls and hospitalisations associated with hypnotics and benzodiazepines often prescribed for sleep disturbance, greater emphasis should be placed on sleep hygiene practices and behavioural interventions. The mechanisms behind the association of sleep and poor cognition are complex and multifactorial. There are several proposed hypotheses. First, insomnia, a common condition among older adults, can cause short term and long term cognitive disruption through immediate impairment of several cognitive domains and having limited important periods of sleep to embed procedural memories [42]. Second, the presence of obstructive sleep apnoea (OSA) leads to fragmented, poor quality sleep, chronic intermittent hypoxia and neuroinflammation which may be contributing factors in the neurodegenerative process underpinning many dementias [43]. Finally, sleep duration is a target to be considered, with Wu et al. demonstrating the lowest incidence of cognitive disorders among individuals sleeping 7-8 h a night compared to those reporting shorter or longer durations of sleep [44]. Given the bidirectional relationship that exists between sleep and cognition, well designed clinical trials are needed to guide management of disordered sleep in order to prevent further decline among those with cognitive impairment.

Within this study we further explored attitudes towards household-level lifestyle interventions, and online modes of intervention, among households. The identified themes are summarised in Table 3. Key information which emerged included the complexity of sleep within households, the willingness to trial dietary interventions, the motivations to participate in physical activity and the openness to digital interventions. With no clinical trial to date targeting the household as the unit of randomisation for lifestyle interventions to delay cognitive decline, the themes which we have identified can be used to inform and plan for future research studies.

**Table 3:** Themes arising from semi-structured interviews

**Themes**	
Household members without dementia were more likely to report poor sleep habits	
Sleep habits were perceived to be much more related to the individual and the household and would be the hardest to change and sustain change within households	
Although most participants had healthy diets, most were interested in making a change if it would be of benefit to cognition	
Participants felt there was a strong link with nutrition and cognition	
Physical activity is challenging to adapt due to lack of motivation and focus when individuals are cognitively impaired and additional barriers to changing lifestyle interventions concentrated around risk of harm	
Motivations for physical activity in households are far beyond strength and cardiovascular benefit; more prominently it used for relaxation and social interaction.	
Digital or online based interventions were appealing with virtual visits highlighted as a method to improve trial participation	
Regular support and reminders would be beneficial to support behavioural change	

The first theme that emerged was that household members without cognitive impairment were more likely to report poor sleep than those with cognitive impairment. There are multiple possible factors involved including caregiver burden, disruption of sleep routine, difficulty falling back asleep after assisting with nocturnal care needs and poor sleep hygiene, such as over-reliance on caffeine, to compensate for this [45]. The complexity of this was highlighted in Household 7, where the person with Lewy Body Dementia had frequent nocturnal visual hallucinations which led to sleep disturbance for his spouse.

Following on from this, a key second theme which emerged was that sleep habits were perceived by most interviewees to be more related to the individual than the household, and that sleep would be the hardest lifestyle habit to change of the three explored during the interviews. Many participants were not aware of non-pharmacological strategies to improve sleep. When provided with a sample online sleep behavioural intervention, participants were uncertain or neutral about its application. There was uncertainty around approaching it as a household rather than as an individual. To date, most RCTs exploring household level interventions targeting sleep have been aimed at families with young children as an intervention to prevent childhood obesity, however, parental or caregiver sleep is rarely measured alongside child sleep habits [46]. Future trials of sleep and cognition should include measurement of sleep of all household members, reasons for disruption, compensation mechanisms alongside objective sleep measurements.

Another theme which emerged was that although most participants had healthy diets, most were interested in making a change and felt there was a strong link with nutrition and cognition. Each household was easily able to volunteer foods that they felt were beneficial for overall brain health. Throughout the interviews it was clear that household members all ate similarly, and if there were dietary restrictions or specific food dislikes for one individual, all members tended to adapt their diet accordingly. Our study supports the literature finding that family and the household environment remains the most continuous factor which influences dietary behaviours [47] and should be considered a key level at which a targeted intervention can be delivered.

Higher income does not always equate to better diets among households. Economic development can lead to increased food security but also lead to higher fat and processed food consumption [48]. Having greater food access and options therefore does not always equate to better nutrition which may have regional implications for population based dietary interventions. The participants in this study did not consider cost or sourcing produce to be a barrier, instead highlighting that recipe ideas or methods to incorporate certain food groups would be of great practical benefit.

Another emergent theme were the challenges relating to changing physical activity behaviours. First, three households commented that it was very challenging to motivate the person with cognitive impairment to exercise. Interestingly, on further exploration, these individuals were very physically active when younger, involved regularly in team sports and took organisational roles within clubs. It has been previously reported that persons with dementia are more likely to abandon recreational and physical activities spaces [49]. Participation in physical activity, particularly group based activities has been shown to be of benefit for cognition [50, 51] but based on our interviews, physical activity interventions need to account for difficulties with focus and attention and not deviate greatly from routine. Many households also expressed that physical injury and risk of falls would be a concern with this type of intervention. Future trials should ensure that physical activity goals are easily incorporated into daily routine, with regular reminders that are achievable for all household members. The incentive for physical activity for most of our interviewees was stress relief and change of environment. Previous individual level trials of physical activity in older persons demonstrated that a targeted custom physical activity intervention was effective in reducing major mobility disability [52] and therefore it is worth investigating if there is benefit for those with cognitive impairment and their households to offset significant functional decline.

A further theme which emerged was that digital interventions were appealing to all households. Very few described challenges with internet access or sensory issues which would adversely impact engagement with online applications. For one individual, this study was the first time he had participated and engaged in a video-based call and found it very user friendly. He stated it had increased his confidence in participating in a future trial incorporating digital technology. Another household promoted the use of virtual trial visits as they were based rurally and attending in person would be time consuming and stressful. Involving patients with dementia in designing and adapting of online interventions is crucial to ensure engagement and acceptability [53]. Given the recent surge in information communication technology, piloting new applications is essential, however, our interviews suggested that many participants were already empowered to use technology, frequently using messaging services and video calling to maintain communication among family members outside of the home. Most had greatest comfort with smartphones and tablets and noted little need for computer or laptop-based activity which should be considered when choosing a digital application host.

An important theme which emerged was the need for regular support and reminders for any household participating in a lifestyle intervention trial. Most concern around participation was due to uncertainty about how and when to incorporate changes. Many felt that reminders or messages about small adaptations on a regular basis would be achievable rather than a single educational seminar at the beginning of the trial. There was a sense among participants that personalising the interventions would allow change to be sustainable and that would allow for better support within households. The use of smart technology to encourage health behaviour change has not been found to be superior to traditional methods following acute myocardial infarction and stroke [54, 55], however, our findings suggest it may have a beneficial role for the cognitively impaired population and their households in allowing a trial intervention to be more feasible.

Given the estimated contribution of lifestyle behaviours to the risk and progression of dementia, and the impact this condition has on household structures, a logical approach would be to target the household as the unit for intervention. Families shape health at an individual level by not only influencing wellbeing at a genetic level, but at an environmental level [56, 57], with members often demonstrating shared behaviours including diet, physical activity and smoking. Despite this, there have been limited studies with households and families as the target unit of randomisation, with most household-level trials in the literature focusing on investigating behavioural change environments for children [12, 58, 59]. By intervening at household level, it is hypothesised that lifestyle adaptations will be better sustained by changing the overall routine and habitual patterns of families. Although adults influence health behaviours of children, there is uncertainty if this would be reflected in other household types, in particular those who are older and may no longer have children living with them.

Observational studies have demonstrated where targeted health interventions (e.g. diet) for cardiovascular risk have been implemented, spouses of participants, who were not themselves enrolled in the study, changed their lifestyle behaviour, suggesting indirect benefits to household members when such interventions are employed by one person [60, 61]. Another population based study demonstrated family status had significant influence over self-reported physical and mental health, with worse outcomes among families who were older, had lower income and lacked availability to insurance [62]. Further investigation is needed to determine the feasibility and effectiveness of lifestyle interventions targeting households with an older age range, where at least one individual has cognitive impairment.

One inherent challenge is the practicality of household recruitment. Our pilot study noted difficulty in recruiting households, as many memory clinic attendees were living independently with family nearby. Definition of a household is of great consideration in trial design and is impacted by local, societal, and cultural factors. A household is traditionally defined as several persons who live in the same dwelling. Consideration should be given to the inclusion of groups of individuals who share the same meals, grocery provision and have physical contact on a consistent and regular basis, most likely living in very close proximity as meeting the definition of a household. Recruitment in certain countries or rural environments may be limited by only including the traditional definition of a household. Further feedback from individuals with cognitive impairment and their caregivers should be used to inform the structure of an eligible household.

### Strengths and limitations

The main strength of this study is the novel use of semi-structured interviews to explore perspectives of all household members on lifestyle intervention trials. Given the lack of trials randomising at a household level, this study provides in-depth insight into the beliefs, preferences, and deterrents of potential participants. Using small semi-structured interviews, rather than larger focus groups, allowed for the person with cognitive impairment and their household members to voice their opinions comfortably and freely. Although other methodological approaches such as surveys provide more structured data, this approach allowed subjects to express a deeper thought process around topics and give commentary on aspects that were of most importance to them.

An additional advantage of conducting the interviews over a web-based video platform was twofold. First, participants were able to have the interviews take place from the comfort of their own home, allowing a relaxed atmosphere and greater open discussion around the relevant topics. Second, this was a method to practically explore digital literacy, ease, and comfort of using online applications among our memory clinic attendees which is relevant for any future clinical trial incorporating technology.

Another strength to this study was the variation in household relationships, with participation from children as well as spouses. This gave additional insights and different perspectives of household members on lifestyle behaviours among different generations, however, we found that diet and physical activity behaviours were similar despite age gaps.

A limitation to this study was identifying suitable participants. Many of our memory clinic attendees live independently supported by family members who do not live within the same household structure. Consideration, in future trials, should be given to the definition of a household; as within West of Ireland populations, many family members live in very close proximity and meals are often shared with regular visits throughout the day, although families may not be living under the same roof. In addition, due to the small sample size, the participants may not be truly representative of our region, however, effort was made to ensure there was a geographical spread ensuring urban and rural differences could be captured.

In addition, a limitation to this study is that we did not explore all lifestyle interventions for the delay of cognitive decline including smoking cessation and reduction in alcohol consumption. The rationale for this was that there is a stronger evidence base for the modification of these lifestyle habits [63, 64] compared to sleep, diet and physical activity which are similar in complexity to measure and to determine optimal dose and require further evaluation in clinical trials.

A further limitation to the study was that we did not show sample digital applications that a trial could use to affect change in diet and physical activity e.g. use of Fitbit tracking application or the Second Nature Programme digital application [65]. An area for future patient and public involvement would be to hold focus groups to discuss and explore appropriate, feasible and acceptable online applications for use by all household participants to inform a trial protocol.

## Conclusions

This limited study identified that sleep, diet and physical activity are justifiable targets for intervention at household level for those affected by cognitive impairment. In addition, there may be cumulative benefits to targeting more than one lifestyle activity in need of intervention further adding to the potential of this type of trial methodology. Barriers to this centre around acceptability of digital intervention, concern around risk of injury and perceptions of sleep behaviour among different household members. Further study with a larger sample of households is needed to explore these findings further, to inform future trial protocols and to maximise study feasibility.

## Data Availability

Relevant extracted data are included within the main body of the manuscript and the tables. Recorded interview transcripts are securely maintained with the authors of the study.

## Abbreviations

RCT: Randomised Controlled Trial
SSI: Semi-structured interview
